# Protection and Damage Repair Mechanisms Contributed To the Survival of *Chroococcidiopsis* sp. Exposed To a Mars-Like Near Space Environment

**DOI:** 10.1128/spectrum.03440-22

**Published:** 2022-12-01

**Authors:** Caiyan Li, Xianyuan Zhang, Tong Ye, Xiaoyan Li, Gaohong Wang

**Affiliations:** a Key Laboratory of Algal Biology, Institute of Hydrobiology, Chinese Academy of Sciences, Wuhan, China; b University of Chinese Academy of Sciences, Beijing, China; University of Minnesota

**Keywords:** *Chroococcidiopsis* sp., Mars-like near space, endurance mechanisms, protection, damage repair

## Abstract

*Chroococcidiopsis* spp. can withstand extremely harsh environments, including a Mars-like environment. However, studies are lacking on the molecular mechanisms of *Chroococcidiopsis* sp. surviving in Mars-like environments. In the HH-21-5 mission, the desert cyanobacterium *Chroococcidiopsis* sp. was exposed to a Mars-like environment (near space; 35 km altitude) for 4 h, and a single-factor environment of near space was simulated on the ground. We investigated the survival and endurance mechanisms of *Chroococcidiopsis* sp. ASB-02 after exposing it to near space by studying its physiological and transcriptional properties. After the exposure, *Chroococcidiopsis* sp. ASB-02 exhibited high cell viability, although photosystem II activity decreased and the levels of reactive oxygen species increased. The single-factor simulation experiments revealed that for the survival of *Chroococcidiopsis* sp. ASB-02 in near space, UV radiation was the most important limiting factor, and it was followed by temperature. The near space environment triggered multiple metabolic pathway responses in *Chroococcidiopsis* sp. ASB-02. The upregulation of extracellular polysaccharides as well as carotenoid and scytonemin biosynthesis genes in response to UV radiation attenuated the extent of radiation reaching the cells. At the same time, genes related to protein synthesis were upregulated in response to the low temperature, overcoming the decrease in metabolic activity that was caused by the low temperature. In near space and after rehydration, the genes involved in various DNA and photosystem II repair pathways were upregulated. This reflected the damage to the DNA and photosystem II protein subunits in cells during the flight and suggested that repair mechanisms play an important role in the recovery of *Chroococcidiopsis* sp. ASB-02.

**IMPORTANCE** This study reported that the protective and repair mechanisms of *Chroococcidiopsis* sp. ASB-02 contributed to its endurance ability in a Mars-like near space environment. In *Chroococcidiopsis* sp. ASB-02, a Mars-like near space environment activated the expression of genes involved in extracellular polysaccharides (EPS), carotenoid, scytonemin, and protein syntheses, which provided additional protection. Additionally, the cell damage repair process enhanced the recovery rate of *Chroococcidiopsis* sp. ASB-02 after the flight. This study will help to enhance the understanding of the tolerance mechanism of *Chroococcidiopsis* sp. and to provide important guidance as to the survival requirements for microbial life in a Mars-like environment.

## INTRODUCTION

Astrobiology is a discipline that emerged with research on the origin of life and the practice of spaceflight. With the rapid development of space technology and the revolutionary progress of modern science, humans have gradually explored the moon, Mars, and other celestial bodies. Significant evidence has proven that among the celestial bodies in the solar system, the environment on Mars is the closest to that on Earth. Therefore, Mars has become the most important target celestial body for studying extraterrestrial life (1). In the 1970s, the United States launched the Viking landers to explore life on Mars. Due to insufficient knowledge of the surface of Mars, conclusive evidence of the existence of life on Mars could not be obtained (2, 3). Some ecological habitats on the Earth are similar to planetary bodies in terms of nutrient composition, biogeochemistry, or topography (4). These places were once considered lifeless because of their harsh environmental conditions; however, the discovery of life in these places now provides new ideas for exploring extraterrestrial life (5). Researchers have conducted some pre-experiments in a simulated Martian environment to study the viability of life as well as the impact of the Martian environment on life. These experiments are expected to provide guidance for identifying new biosignatures and for understanding the evolution and habitability of life on Mars (6).

The simulated Martian environment includes some land simulation stations, ground simulation equipment, and near space, among other qualities. Compared with other simulated Martian environments, the near space aspect offers many advantages. First, the environment of near space is similar to that of the Martian surface environment (7). Second, experiments are more economical in near space than in the International Space Station flight and ground simulations; that is, the flight opportunities are more flexible, and the volumes and weights of payloads can be as large as necessary to satisfy experimental requirements (8). Therefore, near space is the best choice for the simulation of the Martian environment.

Researchers from several countries have conducted life science experiments in near space. The near space environment mainly exhibits low temperature, desiccation, intense ultraviolet (UV) radiation, high-energy ionizing radiation, and low environmental pressure (9, 10). In a study, some fungal and bacterial species were brought to near space using a large scientific balloon and exposed for 5 h. The results revealed that the survival of Aspergillus niger and Salinisphaera shabanensis was not affected by a UV-shielded environment; however, when fully exposed to near space, after exposure to UV radiation, the survival of both was reduced by a 2 and 4 orders of magnitude, respectively. Staphylococcus capitis subsp. *capitis* can only survive in a UV-shielded environment. *Buttiauxella* sp. MASE-IM-9 is the most vulnerable, and exposure to both UV and UV-shielded conditions can have lethal effects on it (11). In another study, three yeast strains were exposed to near space. The results revealed that when the yeast strains were fully exposed to near space, the viability of *Naganishia friedmannii* 16LV2 and *Exophiala* sp. *strain* 15LV1 decreased by 99% and 90%, respectively. However, for *Holtermanniella watticus* 16LV1, only desiccation caused a 99% loss of viability, and complete viability was lost after UV exposure (12). Smith et al. studied the survival of Bacillus subtilis in near space and reported that UV radiation can kill 99.9% of B. subtilis; however, low temperature, low pressure, and desiccation did not exhibit any effect on its viability (13). It can be seen that UV radiation is the main environmental factor that restricts the survival of most organisms in near space. *B. pumilus* SAFR-032 is a strain with the ability to resist UV radiation. This was explained through a comparative genomic analysis by Tirumalai et al., who reported that five new genes related to DNA repair are present in *B. pumilus* SAFR-032 (14). In another study on *B. pumilus* SAFR-032, Khodadad et al. resequenced surviving spores after their exposure to near space, and relatively few nucleotide variations were detected (15). The presence of the DNA repair mechanisms in *B. pumilus* SAFR-032 may account for the fewer number of nucleotide variations. Other studies have explained the survival strategies of organisms in near space at the transcriptome level. During the HH-19-2 mission, the near space environment stimulated the expression of genes related to antioxidant enzymes and DNA repair in algae, which may be the protective mechanism for *Nostoc* sp. to maintain high survival in near space (16). At present, few studies are available on how organisms can survive in near space, and the effect of exposure to near space should be studied on more organisms in the future.

In extreme and arid regions on the Earth, such as the Antarctic Dry Valleys and the Atacama Desert, microscopic fissures, structural cavities, and stone-soil interfaces of rocks are inhabited by *Chroococcidiopsis* sp. (17). *Chroococcidiopsis* sp. is considered a representative of “eoanhydrobiotes”, and all stages of its life cycle undergo anhydrobiosis. Anhydrobiosis refers to some organisms that can survive the loss of all, or almost all, water and enter into a state of suspended animation. In cases of extreme desiccation, *Chroococcidiopsis* sp. can enter a state of suspended animation and can resume metabolism when rehydrated (18). This anhydrobiote is extremely tolerant to desiccation and radiation (19, 20). After 4 years of storage under desiccation, *Chroococcidiopsis* sp. CCMEE029 was observed to survive (21). The resistance of *Chroococcidiopsis* sp. to ionizing radiation ranks second only to Deinococcus radiodurans, and its ability to withstand simulated Mars UV radiation is 10 times that of B. subtilis (22, 23). In addition, *Chroococcidiopsis* sp. can tolerate 13 kJ/m^2^ of UVC radiation and 12 kGy of gamma radiation (22, 24, 25). In view of this, *Chroococcidiopsis* sp. is considered a model system for astrobiological research (19). However, at present, the research on *Chroococcidiopsis* sp. under space conditions mostly focuses on physiological properties. To explore the effects of the near space environment on *Chroococcidiopsis* sp. and its adaptation, in this study, we investigated the physiological changes and the gene expression of *Chroococcidiopsis* sp. under Mars-like near space environmental conditions via a zero-pressure balloon flight.

## RESULTS

### Effects of a near space environment on the physiology of *Chroococcidiopsis* sp. ASB-02.

**FIG 1:**
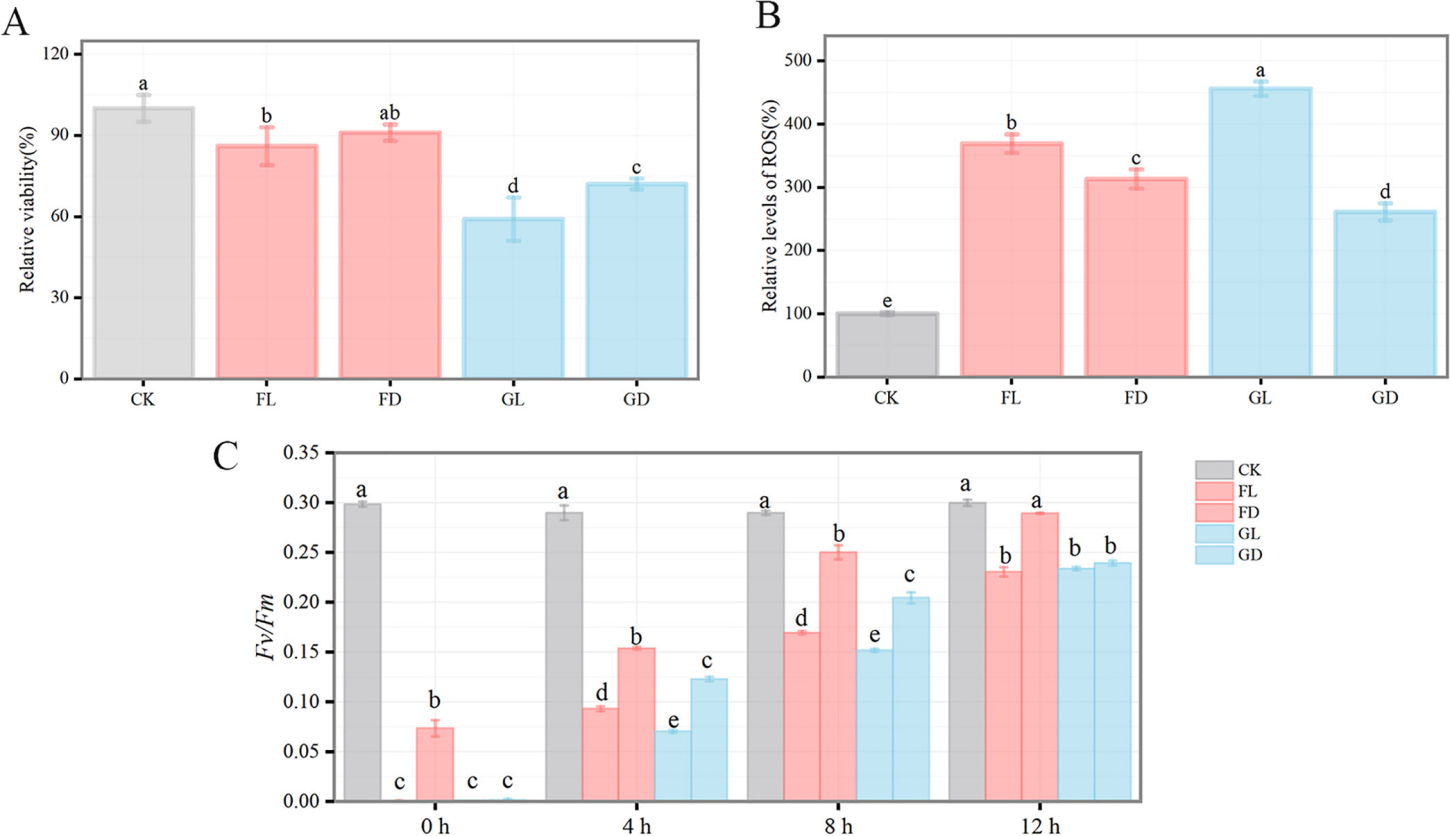
Physiological response of *Chroococcidiopsis* sp. ASB-02 in the flight and ground groups. (A) Cell viability. (B) Relative levels of ROS. (C) PS II activity (*Fv/Fm*) of *Chroococcidiopsis* sp. ASB-02 at 4, 8, and 12 h of rehydration and of the dry sample (0 h). CK indicates normal cultured samples. Bars with different letters indicate statistically significant differences between groups with *P* < 0.05.

**(i) Cell viability.** After exposure to near space for 4 h, the cell viability of the FL group significantly decreased, compared with that of the CK group (Fig. 1A). No significant difference was observed in terms of cell viability between the FD and CK groups (Fig. 1A), indicating that the UV-shielded environment of the near space had no effect on survival. Moreover, we observed that the cell viability of the flight group was significantly higher than that of the ground group (Fig. 1A).

**(ii) Relative levels of reactive oxygen species (ROS).** Compared between the CK and GD groups, ROS production in the flight group increased, and the ROS content in the FL group was 18% higher than that observed in the FD group (Fig. 1B). This indicated that the near space environment increased the level of oxidative stress in *Chroococcidiopsis* sp. ASB-02. The ROS content in the GL group was the highest, at 1.24 and 1.74 times higher than those of the FL and GD groups, respectively (Fig. 1B).

**(iii) Photosystem II (PS II) activity.** After exposure to near space, the *Fv/Fm* of each group was nearly 0 (Fig. 1C). After rehydration, the *Fv/Fm* of the flight and ground groups gradually increased with the increase in the rehydration time. From 4 to 12 h, the *Fv/Fm* of the FL, FD, GL, and GD groups increased by 148%, 88%, 226%, and 95%, respectively (Fig. 1C). The rate of increase of *Fv/Fm* in the light group was higher than that observed in the dark group. After rehydration for 12 h, the *Fv/Fm* of the FD group returned to normal, compared with that of the CK group (Fig. 1C). The *Fv/Fm* of the FL, GL, and GD groups recovered by 80%, 81%, and 83%, respectively (Fig. 1C).

### Effects of a near space single factor environment on the physiology of *Chroococcidiopsis* sp. ASB-02.

**FIG 2:**
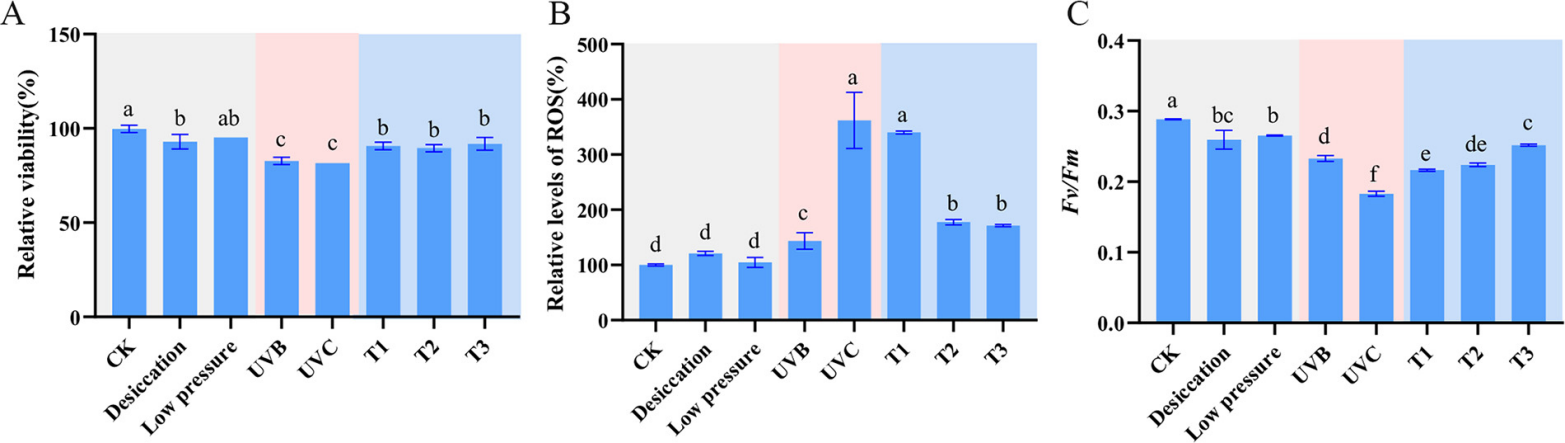
Physiological response of *Chroococcidiopsis* sp. ASB-02 to a near space single factor environment. (A) Cell viability. (B) Relative levels of ROS. (C) PS II activity (*Fv/Fm*). Bars with different letters indicate statistically significant differences between groups (*P* < 0.05). T stands for temperature. T1: −20°C (1 h 35 min), 4°C (30 min), 15°C (20 min), 25°C (20 min), 35°C (20 min), and 45°C (1 h); T2: −20°C (1 h 35 min), 4°C (30 min), 15°C (20 min), and 25°C (20 min); T3: −20°C (1 h 35 min).

The results revealed that low pressure had no effect on the survival, PS II activity, or ROS production. UVB and UVC radiation significantly reduced the viability of cells (Fig. 2A). UVC radiation significantly decreased *Fv/Fm* and increased the ROS content (Fig. 2B and C). Compared with desiccation, temperature had no effect on the cell viability (Fig. 2A); however, temperature fluctuations caused stress to *Chroococcidiopsis* sp. ASB-02 (Fig. 2B and C). In the three temperature ranges, the decrease in *Fv/Fm* mainly occurred in T2 and T3, and no significant difference in *Fv/Fm* was observed between T1 and T2 (Fig. 2C). However, the effect of temperature on the ROS level exhibited an opposite trend. No significant difference was observed in the ROS content between T2 and T3, and the ROS content at T1 was significantly increased, compared with those of T2 and T3 (Fig. 2B).

### Transcriptional changes in *Chroococcidiopsis* sp. ASB-02 in near space and after rehydration.

**FIG 3:**
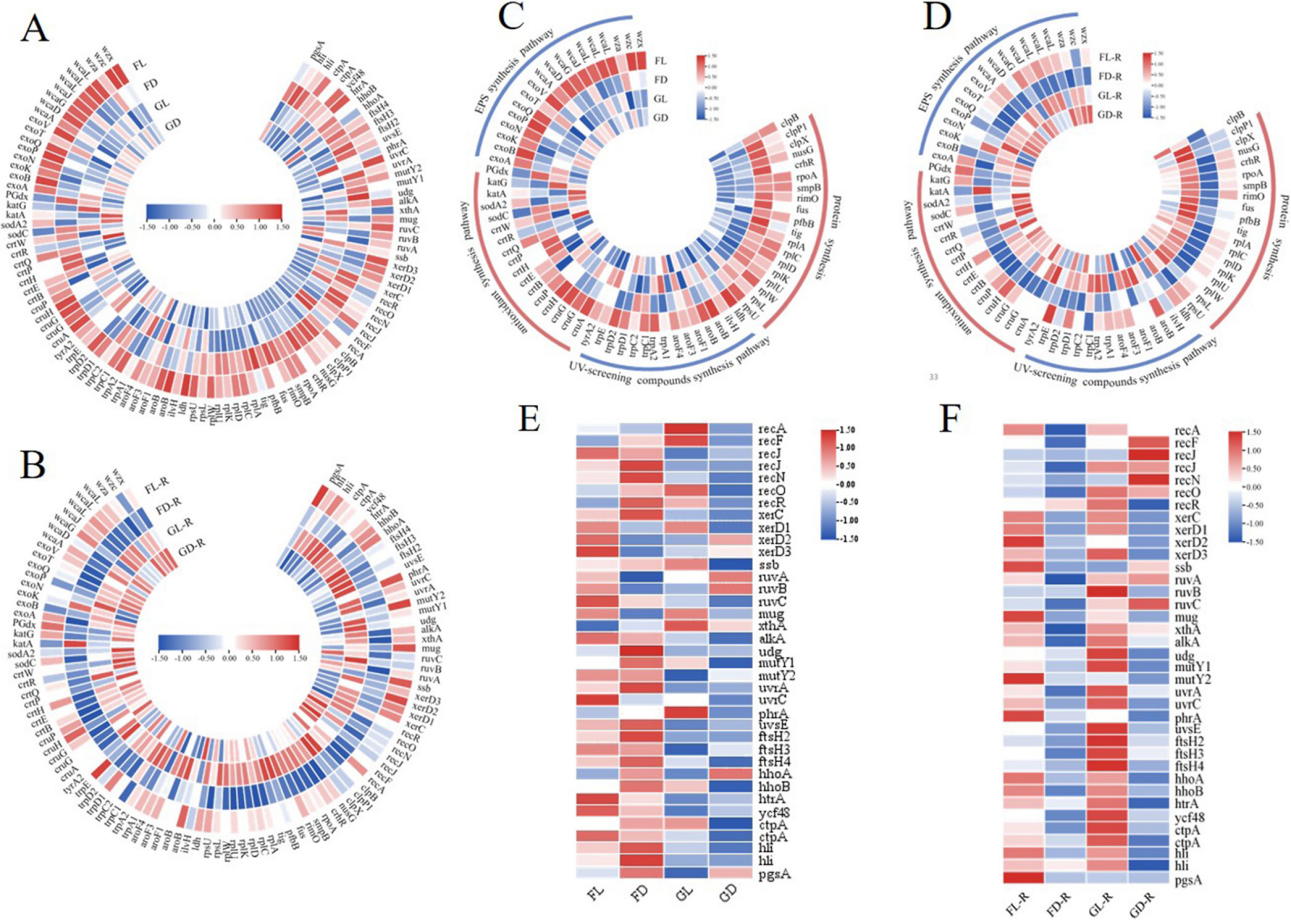
Total transcriptional changes in genes in the flight and ground groups before (A) and after rehydration (B). Transcriptional changes in genes involved in the EPS synthesis pathway, the antioxidant synthesis pathway, the UV-screening compounds synthesis pathway, and protein synthesis in the flight and ground groups before (C) and after rehydration (D). Transcriptional changes in genes involved in the DNA and PS II repair pathways in the flight and ground groups before (E) and after rehydration (F). Red and blue indicate upregulated and downregulated genes, respectively.

**(i) Transcript levels of genes of the extracellular polysaccharides (EPS) synthesis pathway.** The genes involved in the EPS synthesis of succinoglycan (SG) and colanic acid (CA) were significantly upregulated in the FL group. The *exo* and *wca* genes are necessary for SG and CA biosynthesis, respectively. In terms of the synthesis of nucleotide sugars, the UDP-glucose-4-epimerase gene (*exoB*) was significantly upregulated in the FL group. The UDP-pyrophosphorylase gene (*exoN*) was upregulated in the FL group (Fig. 3C). Similarly, the fucose-synthase gene (*wcaG*) was also significantly upregulated in the FL group (Fig. 3C). Glycosyltransferases are responsible for gradually assembling repeat units on the C55 (undecaprenyl diphosphate) linker (26). The gene encoding glycosyltransferase *exoA* was upregulated in the light group, and *wcaA* and *wcaL* were upregulated in the FL group (Fig. 3C). In the periplasmic space, the polymerase that aggregates repeat units is encoded by *exoQ* and *wcaD*, both of which were upregulated in the FL group (Fig. 3C). The polysaccharide copolymerase (PCP) and the outer membrane polysaccharide export protein (OPX) are responsible for the transport of polymeric repeat units from the periplasm to the cell surface (27), with the PCP genes (*exoP* and *wzc*) being upregulated in FL group (Fig. 3C). However, the OPX genes (*exoT* and *wza*) exhibited no significant changes in the FL group (Fig. 3C). After the samples of the flight and ground groups were rehydrated for 12 h, some genes involved in EPS synthesis in the FL-R group exhibited slight upregulation (Fig. 3D).

**(ii) Transcript levels of genes of the antioxidant synthesis pathway.** The expression patterns of the genes associated with nonenzymatic antioxidant-carotenoid synthesis were monitored in the transcriptome data. Compared with the FD group, the gene encoding phytoene synthase (*crtB*) was significantly upregulated in the FL group (Fig. 3C). The *crtQ* gene was upregulated in the flight group (Fig. 3C). The lycopene cyclase gene *cruA* was significantly upregulated in the FL group, and *cruP* was upregulated in the light group (Fig. 3C). The glycosyltransferase gene (*cruG*) and Rieske (2Fe-2S) domain protein gene (*cruH*) were significantly upregulated in the FL group (Fig. 3C). However, the transcript levels of the β-carotene hydroxylase gene (*crtR*) and the beta-carotene ketolase gene (*crtW*) were lower in the FL group than in the FD group (Fig. 3C). Some antioxidant enzyme genes (*sodC*, *katG*, and *PGdx*) were downregulated in the FL group (Fig. 3C). The expression of most genes involved in antioxidant pathways in the FL-R group did not significantly change after the samples of the flight and ground groups were rehydrated; however, the FD-R group exhibited downregulation of these genes (Fig. 3D).

**(iii) Transcript levels of genes of the synthesis pathway of UV-screening compounds.** In the biosynthetic pathway of the UV-screening compound scytonemin, the *trp* and *tyr* genes were upregulated in the FL group (Fig. 3C). The rate-limiting enzyme gene (*aroB*) in the shikimic acid pathway was upregulated in the FL group (Fig. 3C). The leucine dehydrogenase gene (*ldh*) and the acetolactate synthase gene (*ilvH*) were upregulated in the light group (Fig. 3C). After the samples of the flight and ground groups were rehydrated, no significant change was observed in the expression of most genes involved in the biosynthesis of scytonemin in the FL-R group (Fig. 3D).

**(iv) Transcript levels of genes involved in protein synthesis.** Genes involved in translation were activated in the flight group. The 50s ribosomal protein gene (*rpl*) and the 30s ribosomal protein gene (*rps*) were upregulated in the flight group (Fig. 3C). The ribosome trigger factor gene (*tig*) and the GTP-binding protein TypA/BipA homolog gene (*fus*) were significantly upregulated in the FD group (Fig. 3C). The caseinolytic protein genes (*clpP1*, *clpB*, and *clpX*) were upregulated in the flight group (Fig. 3C). The transcription antitermination protein gene (*nusG*), RNA helicase gene (*crhR*), and RNA polymerase alpha subunit gene (*rpoA*) involved in transcription and RNA maintenance were upregulated in the flight group (Fig. 3C). The expression of genes involved in protein synthesis in the FL-R group did not significantly change after the samples of the flight and ground groups were rehydrated; however, the FD-R group exhibited downregulation (Fig. 3D).

**(v) Transcript levels of genes involved in the DNA repair pathway.** The genes involved in homologous recombination (HR) exhibited various expression patterns in the FL and FD groups (Fig. 3E). The expression levels of some genes involved in the RecF pathway (*recJ*, *recN*, *recO*, and *recR*) increased in the FD group (Fig. 3E). Excision repair can remove damaged bases by two approaches: base excision repair (BER) and nucleic acid excision repair (NER). Several DNA glycosylase genes (*mug*, *alkA*, and *mutY2*) in the BER pathway were upregulated in the FL group, and the *alkA*, *udg*, *mutY1*, and *mutY2* genes were upregulated in the FD group (Fig. 3E). The excision nuclease genes, namely, *uvrA* and *uvrC*, involved in the NER pathway were upregulated in the FL group. The *uvsE* gene was upregulated in the flight group (Fig. 3E). When the samples of the flight and ground groups were rehydrated, the genes involved in DNA repair were downregulated in the FD-R group. The genes involved in the RecF pathway were downregulated in the FL-R group. Most DNA glycosylase genes, *uvrA*, and *uvrC* exhibited weak upregulation in the FL-R group. However, the main factor of photoreactivation, namely, *phrA*, was significantly upregulated in the FL-R group (Fig. 3F). In addition, the results revealed that most of the genes involved in DNA repair were upregulated in the GL-R group, compared with the FL-R group (Fig. 3F).

**(vi) Transcript levels of genes involved in the PS II repair pathway.** During PS II repair, the *ftsH* gene involved in the degradation of damaged D1 protein exhibited weak upregulation in the FL group (Fig. 3E). The *htrA*, and *ycf48* genes encoding the PS II assembly protein and the *ctpA* gene encoding the C-terminal D1 processing protease were upregulated in the FL group (Fig. 3E). The genes involved in PS II repair were upregulated in the FD group (Fig. 3E). When the samples of the flight and ground groups were rehydrated, *ftsH* was downregulated in the FL-R group, and the cyanobacterial Deg-type protease genes (*hhoA*, *hhoB*, and *htrA*) were upregulated (Fig. 3F). The *hli* gene encoding the high-light-induced proteins and the *pgsA* gene involved in the biosynthesis of phosphatidylglycerol were upregulated in the FL-R group (Fig. 3F). The genes involved in PS II repair were downregulated in the FD-R group (Fig. 3F).

## DISCUSSION

### Survival of *Chroococcidiopsis* sp. ASB-02 after exposure to near space.

**FIG 4:**
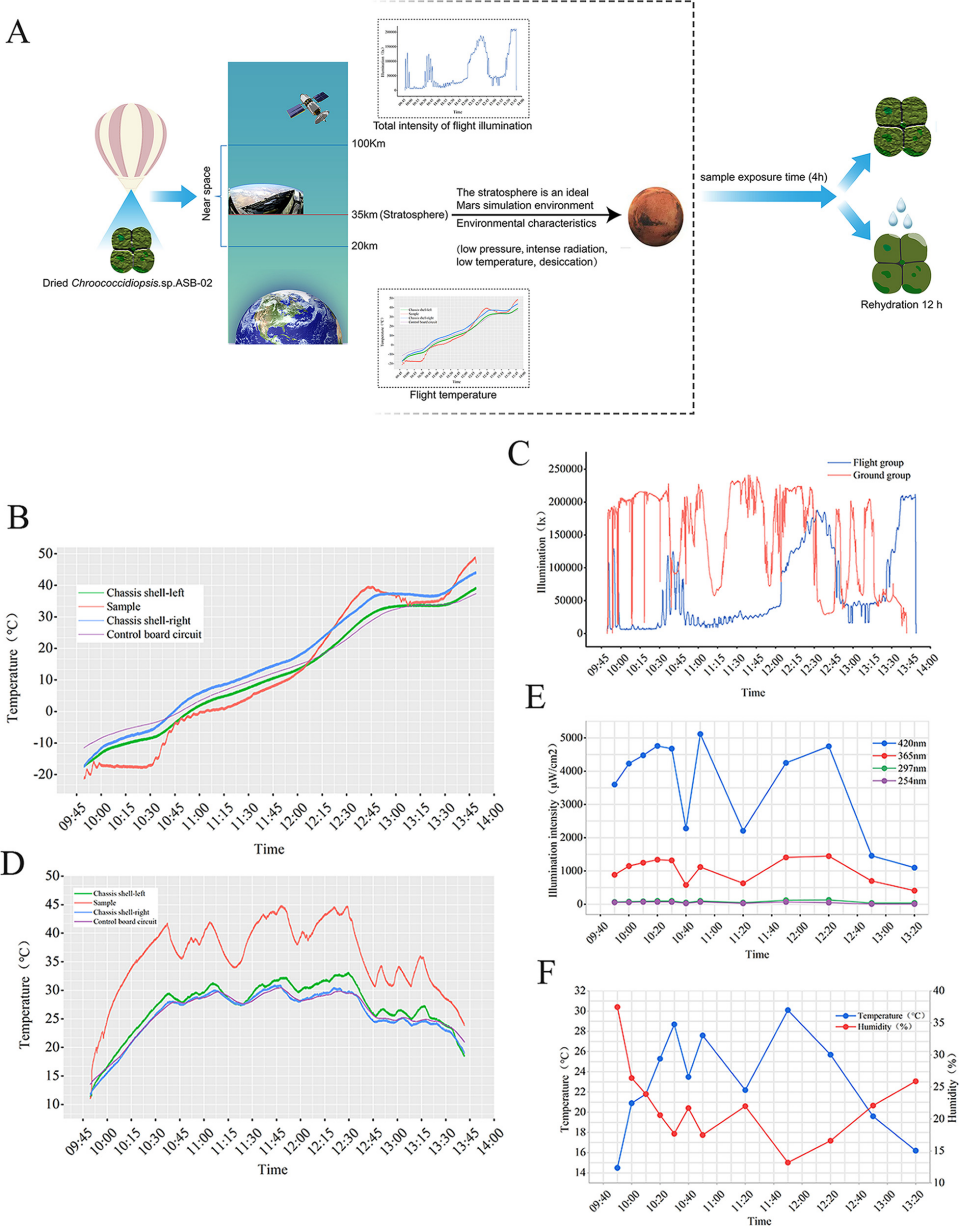
Flight experiment process and monitoring of changes in the near space and ground environments. (A) HH-21-5 mission flight with *Chroococcidiopsis* sp. ASB-02 exposed to near space (simulated Mars-like environment). (B) Flight temperature. (C) Total intensity of flight and ground illumination (these data are only used for qualitative analysis). (D) Ground comparison test temperature. (E) Ground environmental illumination intensity. (F) Ground environmental temperature and humidity.

During the HH-21-5 mission, we investigated the survival of the *Chroococcidiopsis* sp. ASB-02 flight in near space. Samples from the flight group exhibited better survival rates (>85%) (Fig. 1A). Their survival rate was better than those of some algae (*Chlorella* sp. and *Nostoc* sp.), extremophilic yeasts (*Naganishia friedmannii* 16LV2, *Exophiala* sp. 15LV1), and the radiotolerant strain *B. pumilus* SAFR-032 (12, 15, 16, 28). Under laboratory and space conditions, *Chroococcidiopsis* sp. is reported to have significant radiation tolerance (19). However, the survival rate of *Chroococcidiopsis* sp. ASB-02 was lower when it was exposed to ground solar radiation. Similarly, the same situation occurred in the GD group. We believe this phenomenon may be due to the high temperature. According to the temperature curve of the ground biological exposure device, it can be seen that the ground group samples were kept at a high temperature (35 to 45°C) environment for more than 2 h during the entire exposure experiment (Fig. 4D). However, the flight group samples were kept at a high temperature environment for less than 1 h (Fig. 4B). Mehda et al. reported that *Chroococcidiopsis* sp. could tolerate the temperature of 35°C; however, the survival of *Chroococcidiopsis* sp. was affected at 40°C (29). In our study, short-term high temperature stress did not affect the survival of *Chroococcidiopsis* sp. ASB-02 (Fig. 1A). However, when the samples were directly exposed to high temperature, a relatively long time of high temperature stress may have led to the loss of some cell viability in the ground group. In addition, considering that UV radiation is the most critical factor affecting the survival of microorganisms in near space, in this study, compared with the FD group, UV radiation did not significantly affect cell viability, reflecting the high resistance of *Chroococcidiopsis* sp. ASB-02 (Fig. 1A).

In this study, we measured the chlorophyll fluorescence parameter *Fv/Fm*, an index of PS II photosynthetic activity, to reflect the influence of a near space environment on the photosynthetic performance of *Chroococcidiopsis* sp. ASB-02 (30, 31). After exposure to near space, *Chroococcidiopsis* sp. ASB-02 PS II activity was barely detected. After rehydration, PS II activity exhibited a recovery trend with increasing rehydration time (Fig. 1C). These results suggested that the near space environment did not cause irreversible damage to PS II and that *Chroococcidiopsis* sp. ASB-02 protected cells from the near space environment by shutting down photosynthetic activity. This result is consistent with a study on *Circinaria gyrosa*, whose PS II activity was not observed in a Mars-like environment but could be effectively recovered after rehydration (32). Although nonfatal damages were still observed, the recovery of PS II activity in *Chroococcidiopsis* sp. ASB-02 in the FL group at the early stage of rehydration exhibited a significant lag compared that of the dark group. Single factor simulation experiments demonstrated that UVC had the greatest effect on PS II. Excessive ROS production in *Chroococcidiopsis* sp. ASB-02, which can be generated by UVC radiation, can affect the PS II reaction center (33). However, high temperature also induced excessive ROS production but did not significantly affect PS II activity (Fig. 2B and C). *Chroococcidiopsis* sp. ASB-02 may adapt to the initial temperature fluctuation and may have developed a protective mechanism by which to avoid the effect of short-term high temperature on PS II.

### Protection in *Chroococcidiopsis* sp. ASB-02 exposed to near space.

UV radiation is the main damaging factor that affects the survival of *Chroococcidiopsis* sp. ASB-02 in near space. In view of the extreme UV radiation in near space, *Chroococcidiopsis* sp. ASB-02 activates three defense mechanisms, namely, EPS, nonenzymatic antioxidants, and UV-screening compounds, to protect cells from fatal effects. EPS is the first line of defense for cyanobacteria to overcome excessive UV radiation (34). The expression levels of EPS biosynthesis genes in *Chroococcidiopsis* sp. ASB-02 were high when exposed to near space UV radiation (Fig. 3A). A thick EPS sheath can increase the effective path lengths of absorbing radiation and can attenuate the level of UV radiation reaching the cells (35). Studies have reported that EPS can screen 67% of UVA, 69% of UVB, and 87% of UVC from transmitting (36). The rest of the UV radiation bypasses the EPS and reaches the cells, and the nonenzymatic antioxidant biosynthesis in the antioxidant system is activated.

Antioxidant systems are the second line of defense for cyanobacteria to overcome the harmful effects of excessive UV radiation (34). Carotenoids are nonenzymatic antioxidants that play an antioxidant role by acting as a sunscreen, dissipating excess light energy, quenching singlet oxygen, and scavenging free radicals (37). Exposure to near space UV radiation may change the expression of genes involved in carotenoid biosynthesis in *Chroococcidiopsis* sp. ASB-02 (Fig. 3C). Phytoene synthase (*crtB*) and ζ-carotene desaturase (*crtQ*) are the enzymes required for lycopene synthesis, and their transcript levels were increased in the FL group. Lycopene synthesis is a key step in carotenoid biosynthesis, and it can lead to the production of many types of carotenoids. The upregulation of this pathway not only increases the production of carotenoids but also provides the precursors required for enhanced photoprotection (38). Furthermore, lycopene cyclase genes (*cruA* and *cruP*), which catalyze the conversion of lycopene into various types of carotenes, were upregulated in response to near space radiation. Transcriptome data revealed that genes (*cruG* and *cruH*) necessary for the synthesis of myxoxanthophyll and synechoxanthin, respectively, were upregulated in the FL group. However, the beta-carotene hydroxylases gene (*crtR*) involved in converting β-carotenoids into zeaxanthin was downregulated in the FL group. The beta-carotene ketolase gene (*crtW*) involved in converting β-carotenoids to echinenone exhibited decreased expression levels in the FL group. These results suggest a shift in the direction of carotenoid production toward myxoxanthophyll and synechoxanthin under strong UV radiation. Myxoxanthophyll is the main pigment in the outer membrane (39). Among carotenoids, myxoxanthophyll has the highest unsaturation and strongest quenching effect on singlet oxygen and other ROS. Myxoxanthophyll acts as a UV photoprotector bound to the outer membrane. When *Chroococcidiopsis* sp. ASB-02 is stressed by UV radiation, myxoxanthophyll can initiate a rapid SOS response before the synthesis of extracellular UV-screening substances (40). However, antioxidant enzymes did not play a role in the resistance of *Chroococcidiopsis* sp. ASB-02 to UV-radiation-induced ROS in this study. Therefore, upregulation of myxoxanthophyll biosynthetic genes in response to near space UV radiation may be the main way by which *Chroococcidiopsis* sp. ASB-02 eliminates ROS.

UV-screening compounds are the third line of defense for cyanobacteria to overcome the harmful effects of excessive UV radiation (41). The expression levels of the major scytonemin biosynthesis genes were mainly increased in the FL group (Fig. 3C). Scytonemin is located in the EPS sheath of cyanobacteria, and it has a complex ring structure that gives it a unique UV absorption pattern (42). Tryptophan and tyrosine are important precursors in scytonemin biosynthesis (43, 44). The tryptophan biosynthesis gene (*trp*) and tyrosine biosynthesis gene (*tyr*) were upregulated when *Chroococcidiopsis* sp. ASB-02 was exposed to near space UV radiation. Aromatic amino acids are the building blocks required for the biosynthesis of scytonemin, whereas shikimic acid is a metabolite required for the synthesis of aromatic amino acids (45–47). The regulatory enzyme gene (*aroG*) and the rate-limiting enzyme gene (*aroB*) are two common genes in the shikimate biological pathway, and both can promote or control the overall rate at which the shikimate pathway adapts to the amount of precursors required in scytonemin biosynthesis under different UV radiation conditions (45). Our results revealed that *aroB* expression was significantly increased in the FL group, whereas *aroF* gene (AroF and AroG are isozymes) expression was relatively low. The possible reason for this is that the high expression of the rate-limiting enzyme AroB will feedback-inhibit the regulatory enzyme AroF to increase the production of shikimate (48).

The temperature changes (low to high) during this flight had some effect on *Chroococcidiopsis* sp. ASB-02, considering that near space is a low temperature environment. Therefore, we focused on the response of *Chroococcidiopsis* sp. ASB-02 to low temperature. After the short-term exposure of *Chroococcidiopsis* sp. ASB-02 to low temperature, we identified some genes that responded to low temperature stress and are involved in transcriptional and translational processes (Fig. 3C), including a DEAD box family RNA helicase gene (*crhR*) that is involved in catalyzing the ATP-dependent unwinding of RNA secondary structures and the annealing of cRNA strands (49). In a low temperature environment, the *crhR* gene not only helps cells overcome the translational obstruction caused by low temperature but also participates in the energy redistribution and photosystem stoichiometry regulation induced by low temperature (50, 51). Caseinolytic proteases (Clps) as cold shock chaperones and proteases play important roles in biological adaptation to low temperature environments (52). The *clpB* gene maintains the activity of PS II complexes at low temperature and helps to enhance the adaptation of cells to low temperature conditions in terms of proliferation and survival (53). Therefore, *Chroococcidiopsis* sp. ASB-02 cells maintained high viability and PS II activity under low temperature conditions (Fig. 2A and C). In addition, many genes encoding ribosomal proteins were upregulated in the flight group. The exposure of prokaryotes to low temperature environments will reduce translation activity, and the enhancement of ribosomal protein genes will help the translation device adapt to low temperatures (50). These results suggest that the cells of *Chroococcidiopsis* sp. ASB-02 were protected from the low temperatures via the enhancement of transcription and translation mechanisms.

### Damage repair process in *Chroococcidiopsis* sp. ASB-02 after exposure to near space and after rehydration.

After exposure to near space, some genes involved in repair pathways were upregulated in the flight groups, particularly in the FD group. After rehydration, the genes involved in repair pathways still exhibited upregulation in the FL-R group, whereas those involved in repair pathways were downregulated in the FD-R group (Fig. 3E and F). The results indicated that both near space environment conditions and rehydration can stimulate the repair effect. When exposed to near space, the repair effect is greater than the damage effect. It may mainly repair the damage caused by high temperatures and minimize the accumulation of damage caused by high temperatures. During the rehydration process, the damage caused by UV radiation is mainly repaired. Studies on the roles of the DNA repair pathways in the recovery of *Chroococcidiopsis* sp. ASB-02 after exposure to a Mars-like environment mainly focused on rehydration (54–56). UV radiation can cause various types of DNA damage. Among the various types of DNA damage, double-strand breaks (DSBs) are the most severe form of damage. They can affect two DNA strands and can even lead to a loss of genetic material (57). HR is one of the major DNA repair mechanisms that uses the RecF pathway to efficiently repair DSBs (58). Several studies have reported that *Chroococcidiopsis* sp. CCMEE 029 repairs DSBs induced by vacuum, prolonged drying, and high-LET radiation conditions by upregulating the expression of genes involved in the RecF pathway after rehydration (54, 55). In our results, the expression of genes involved in the RecF pathway was low in the FL and FL-R groups, indicating a lack of DSBs induced by UV radiation in the samples from this flight. However, most of the genes involved in the BER pathway were upregulated, suggesting that the BER pathway repairs DNA damage induced by near space UV radiation. This may be related to the high production of ROS under UVC radiation. Indeed, UV radiation typically damages DNA by inducing excess ROS production in cells or by a direct transfer of radiant energy (59). Photoreactivation, UV damage endonuclease (UvsE)-dependent excision repair (UVER), and NER are well-known for repairing DNA damage induced by UV radiation (60). Our results revealed that after rehydration, DNA photolyase plays a major role in repairing cyclobutane pyrimidine dimers caused by UV radiation. Related studies on *Chroococcidiopsis* sp. CCMEE 029 are available (56).

In addition to repairing DNA lesions, the low PS II activity of *Chroococcidiopsis* sp. ASB-02 gradually recovered after the flight with rehydration (Fig. 1C). Cyanobacteria maintain the normal function of PS II through a repair cycle. The damaged subunits trigger a structural change in the dimeric PS II complex to form a monomeric RC47 complex that contains the damaged subunits. This structural change allows either FtsH or Deg protease to participate in the degradation of the damaged subunits (61). Our study reported that only three Deg homolog genes were significantly upregulated in the FL-R group (Fig. 3F). Some studies have reported that in cyanobacteria, *hhoA* is upregulated twofold during the repair of PS II damage caused by UV/light stress. The upregulation of the Deg homolog genes plays a photoprotective role but is not crucial for the degradation of damaged subunits (62, 63). However, the FtsH complex can bind to the N terminus of the damaged subunits, drive the removal of the damaged subunits via ATP hydrolysis, and exhibit a high rate of degradation in the Zn^2+^ protease domain (61). The *ftsH* gene exhibited weak upregulation only in the FL group (Fig. 3E), suggesting that the degradation of a small number of PS II protein subunits occurs upon near space exposure. Phosphatidylglycerol, Ycf48, and CtpA are critical in the assembly of PS II (64). In addition, Ycf48 can selectively replace damaged D1 protein during PS II repair (65). The *ycf48* and *ctpA* genes are involved in PS II assembly when exposed to near space conditions, and the *pgsA* gene is involved in PS II assembly after rehydration. The upregulation of genes involved in PS II assembly indicated that *Chroococcidiopsis* sp. ASB-02 attempted to repair the effects of the near space environment on PS II by promoting the generation of new subunits and the dynamic assembly of PS II. Furthermore, during PS II repair, since chlorophyll molecules hinder D1 protein degradation, chlorophyll molecules must be “cleared” to ensure that the D1 polypeptide chain can be pulled out of the membrane. After rehydration, *Chroococcidiopsis* sp. ASB-02 promoted the binding of high-light-induced proteins to chlorophyll by activating the expression of *hli* and temporarily storing chlorophyll (66). PS II damage and repair exist in a dynamic balance. The upregulation of PS II repair genes after near space exposure and rehydration increased the activity of the PS II repair cycle, making repair efficiency greater than damage efficiency in order to meet the requirements of normal PS II activity. This is consistent with the rapid recovery rate in PS II activity in the FL-R group during rehydration.

### Conclusions.

**FIG 5:**
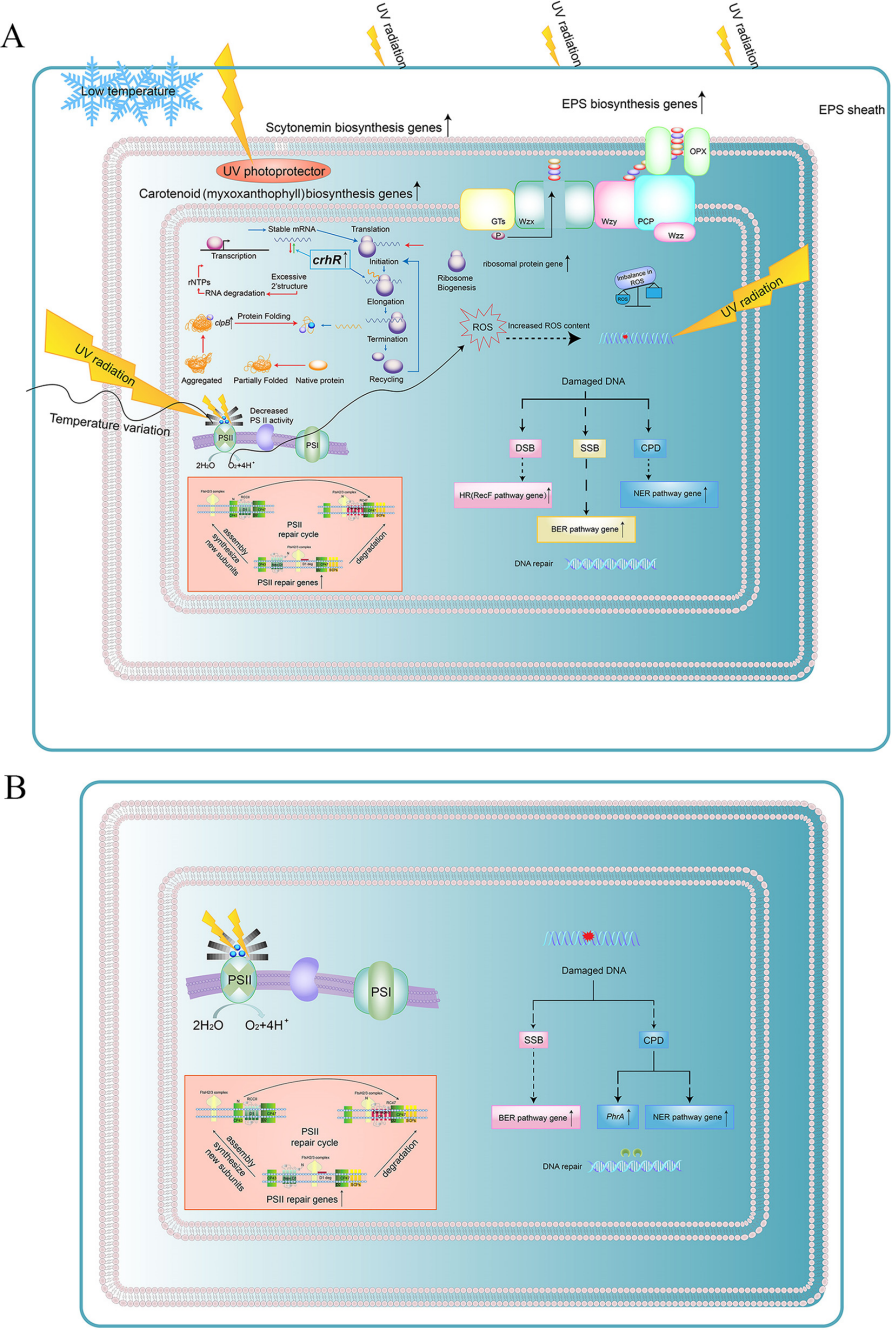
(A) Schematic diagram of the protection and damage repair process in *Chroococcidiopsis* sp. ASB-02 exposed to near space. (B) Schematic diagram of the damage repair process in *Chroococcidiopsis* sp. ASB-02 after rehydration.

Understanding the endurance mechanisms of *Chroococcidiopsis* sp. to extreme environments is an important step in applying it in space exploration. To the best of our knowledge, this is the first study to assess transcriptome levels and reveal the protective and repair mechanisms adopted by *Chroococcidiopsis* sp. to enhance its endurance to a Mars-like environment. *Chroococcidiopsis* sp. ASB-02 protects cells from lethal damage caused by near space UV radiation or low temperatures by activating the expression of genes involved in EPS as well as carotenoid, scytonemin, and protein syntheses (Fig. 5A). The active cell damage repair mechanism effectively repaired the DNA and PS II damage in near space, thereby ensuring the recovery of *Chroococcidiopsis* sp. ASB-02 to its normal state (Fig. 5A and B). The formation of a protection-repair mechanism and the continued occurrence of repair after rehydration may enable *Chroococcidiopsis* sp. to survive in a Martian environment for a long time and to become a pioneer species in Mars exploration.

## MATERIALS AND METHODS

### *Chroococcidiopsis* culture and sample preparation.

*Chroococcidiopsis* sp. ASB-02 was isolated from the desert of Urad Middle Banner, Bayan Nur City, Inner Mongolia Autonomous Region, China, and it is currently stored at the Group of Algae Stress Physiology and Space Biology, Institute of Hydrobiology, Chinese Academy of Sciences. *Chroococcidiopsis* sp. ASB-02 was cultured in BG-11 medium at 25°C, and the light intensity was 40 μmol m^−2^ s^−1^ with a light dark cycle of 16 h/8 h (24, 67).

**FIG 6:**
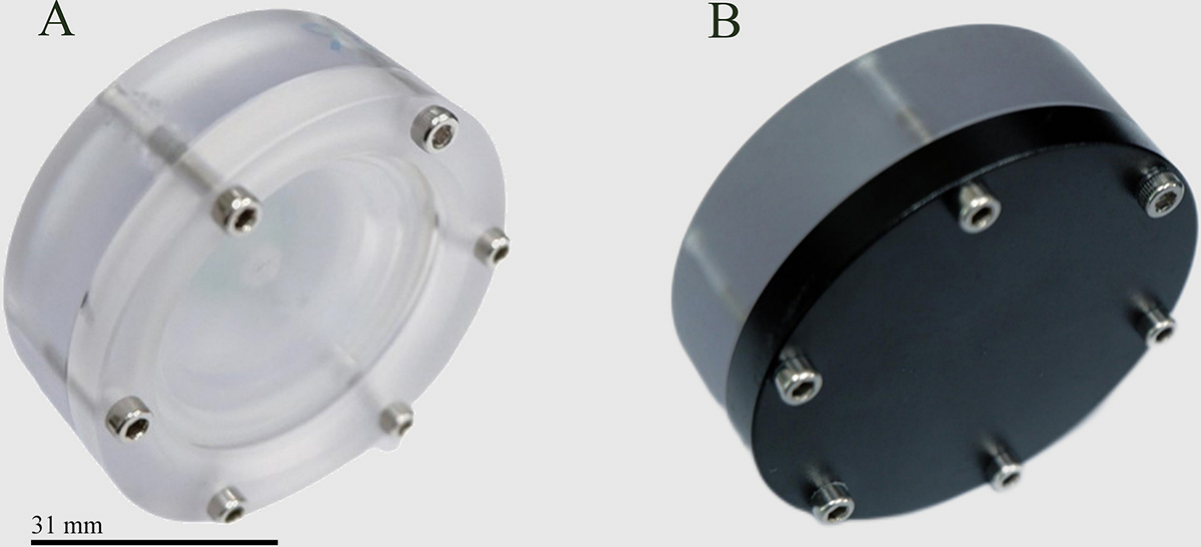
Sample containers for the (A) light groups (FL and GL) and (B) dark groups (FD and GD).

Cells in the logarithmic growth phase were subjected to centrifugation. The obtained cell pellet was resuspended to obtain a density of approximately 10^10^ cells/mL. The same amount of algae was taken and evenly added to the substrate (surface area of the substrate was 12.56 cm^2^), which was then covered with 3 mL BG-11 agar medium in the sample container (surface area of the container was 110.97 cm^2^) and placed on a clean bench for drying. Overall, 32 sample containers were divided into four groups: the flight group (including the flight light group [FL group] and the flight dark group [FD group]) and the ground control group (including the ground light group [GL group] and the ground dark group [GD group]). The sample containers of the FL and GL groups were equipped with UV-grade quartz glass that could allow UV radiation (≥85%) to pass through (Fig. 6A), and those of FD and GD groups were equipped with black aluminum covers that could block the transmission of UV radiation (Fig. 6B). The airtight type container had a hermetic seal ring, and the leak rate of the container was ≤10^−5 ^Pa·m^3^/s under a pressure of one standard atmosphere.

### Flight experiment process and sample processing.

HH-21-5 biological exposure flight experiment was conducted in Dachaidan, Qinghai Province, on September 17, 2021. Before the balloon flight, the sample containers were installed onto the Biological Sample Exposure Payload, which was equipped with an electronically manipulated cover to control the exposure time. The zero-pressure balloon was inflated, and the balloon was released in the launching base (37.7427961°N, 95.3401969°E). When the balloon reached 35 km, it began to fly flat, after which the cover of the Biological Sample Exposure Payload was opened (Fig. 4A). At this time, the exposure experiment and the ground control experiment were initiated. The flight lasted for 7 h, including 4 h of sample exposure, and the Biological Sample Exposure Payload landed at a nearby site (38.1884107°N, 94.8852912°E) (27). The environmental changes of the near space and ground environments were monitored during the experiment (Fig. 4).

After the flight, the samples were placed in an ice box and brought back to the laboratory for analysis. The 8 samples from the FL group were divided into three parts. One part was used to measure physiological indexes. A second part, RNAlater, was directly added to stabilize the RNA in the desiccated samples for further RNA sequencing. The remaining part was rehydrated (12 h) and further used for RNA sequencing. Each part is guaranteed to be three replicates. The sample treatment methods for the FD, GL, and GD groups were the same as above.

### Physiological index determination.

**(i) Cell viability.** CellCounting-Lite 2.0, a cell viability detection reagent based on a luciferase system, was used to determine the cell viability of samples. CellCounting-Lite 2.0 was added to each sample, and the cells were fully lysed by shaking and mixing. The cells were placed at room temperature for 10 min, and the luminescence was detected using a multifunction measuring instrument (Filter Max F5, Molecular Devices). Cell viability was expressed in terms of relative viability (%).

### Reactive oxygen species.

The ROS content in the samples was determined using a Reactive Oxygen Species Assay Kit (Beyotime Institute of Biotechnology, Haimen, China). The fluorescent probe DCFH-DA was loaded into the cells, and the fluorescence of DCF was detected at an excitation wavelength of 488 nm and an emission wavelength of 525 nm. The level of intracellular ROS was reflected by DCF fluorescence. The ROS levels were expressed in terms of relative levels of ROS (%).

### PS II activity.

The sample was rewetted with BG-11 medium and placed at 25°C under 40 μmol m^−2^ · s^−1^ light. The chlorophyll fluorescence parameters of the sample were determined using a Plant Efficiency Analyzer (Handy PEA, Hansatech, UK) at 0, 4, 8, and 12 h. The fluorescence parameter was determined using the formula *Fv/Fm* = (*Fm*-*F0*)/*Fm*, where *Fv* represents the variable fluorescence yield, *Fm* represents the maximum fluorescence yield after the saturation pulse, and *F0* represents the minimum fluorescence in the dark (24). The adaptation time of the samples to the dark was 15 min.

### RNA sequencing.

The samples from the FL, FD, GL, and GD groups were mixed with RNAlater and rehydrated for 12 h (FL-R, FD-R, GL-R, and GD-R). Further, they were sent to Shanghai Majorbio Bio-pharm Technology Co., Ltd., for RNA sequencing. The total RNA of each sample was extracted using the TRIzol reagent (Invitrogen, Carlsbad, USA), and the concentration and purity of the RNA were detected using a NanoDrop 2000. The integrity of the RNA was detected using agarose gel electrophoresis (68). Then, rRNA was removed from the total RNA using a Ribo-Zero rRNA Removal Kit (Epicentre, Madison, USA). The mRNA was randomly broken into short fragments of about 200 bp in length and was transcribed into the first-strand cDNA under the action of reverse transcriptase. The second-strand cDNA was synthesized subsequently using RNase H and DNA polymerase I, with dUTP being used instead of dTTP in the dNTPs reagent to form the base. Before PCR amplification, the second-strand cDNA was digested by the UNG enzyme so that the library contained only the first-strand cDNA. Then, the cDNA was enriched and amplified via polymerase chain reaction (PCR) for 15 cycles. The PCR products were isolated using Certified Low Range Ultra Agarose, and the appropriate fragments were selected for sequencing on an Illumina HiSeq 2500 platform (69).

The reads that contained low quality bases, adapter sequences, and 10% N (undefined bases) were removed from the raw reads to obtain clean data. The Bowtie software package was used to compare the clean data with the genome of *Chroococcidiopsis* sp. ASB-02 (unpublished). The expression levels of genes were calculated using TPM (transcripts per kilobase million) values.

### Single factor simulation experiment of the near space environment.

To understand the effects of UV radiation, low pressure, and temperature on *Chroococcidiopsis* sp. ASB-02 in near space, we conducted a single-factor simulation experiment in the laboratory. Because of the lack of UV radiation data that can be used for quantitative analysis and the lack of records of pressure during the flight, we referred to the data at an altitude of 36 km (UVB, 1000 μw/cm^2^; UVC, 260 μw/cm^2^; and pressure, −14 lb/in^2^) (7). The temperature changed considerably during this flight, from −20°C to 50°C (Fig. 4B). Therefore, we set three different temperature treatments (T1, T2, and T3) to explore the effect of temperature on the cells at various stages: T1: −20°C (1 h 35 min), 4°C (30 min), 15°C (20 min), 25°C (20 min), 35°C (20 min), and 45°C (1 h); T2: −20°C (1 h 35 min), 4°C (30 min), 15°C (20 min), and 25°C (20 min); T3: −20°C (1 h 35 min).

### Statistical analyses.

The statistical data were processed using Microsoft Office Excel. The results were expressed as the mean ± standard deviation (SD). Statistical significance was analyzed using a one-way analysis of variance in the SPSS 23.0 software package. A *P* value of <0.05 was considered to be indicative of a statistically significant result.

### Data availability.

The key gene expression data of the flight and ground group samples after their exposure to near space and rehydration have been added to the supplemental material (Table S1).The transcriptome sequencing raw data have been deposited in the National Center for Biotechnology Information Sequence Read Archive (NCBI SRA) database (accession number: PRJNA900729).
