# Supplementary material for: Protection and Damage Repair Mechanisms Contributed To the Survival of Chroococcidiopsis sp. Exposed To a Mars-Like Near Space Environment
Source: Microbiol Spectr. 2022 Dec 1;10(6):e03440-22. doi: 10.1128/spectrum.03440-22 (PMC9769825; doi:10.1128/spectrum.03440-22)
Supplement: Supplemental file 1 — Table S1. Download spectrum.03440-22-s0001.pdf, PDF file, 0.08 MB [file spectrum.03440-22-s0001.pdf]

Gene	GD	GL	FD	FL	GD-R	GL-R	FD-R	FL-R
pgsA	17.08	15.01	17.59	16.17	25.93	26.15	26	30.67
hli	367.62	387.29	839.67	537.6	530.43	1215.1	945.08	1058.86
hli	3704.25	4104.78	5053.01	4534.18	46.51	72.92	52.08	76.88
ctpA	1.26	1.76	2.26	2.66	24.37	78.18	37.32	51.22
ctpA	4.36	10.68	10.32	8.06	6.44	10.77	5.79	7.95
ycf48	16.1	14.7	18.31	20.09	38.29	61.93	31.87	44.2
htrA	0.19	0.14	0.28	0.41	0.1	0.5	0.29	0.4
hhoB	5.79	9.5	11.08	8.43	5.34	8.53	4.58	8.57
hhoA	5.4	4.61	5.29	4.73	7.07	9.75	7.8	10.29
ftsH4	72.93	84.11	102.71	92.55	51.95	109.62	53.6	62.91
ftsH3	72.78	63.51	81.14	82.55	336.25	470.33	268.4	340.93
ftsH2	105.04	105.2	113.75	109.6	207.64	250.88	195.5	208.64
uvsE	116.84	109.32	149.82	137.18	66.08	74.22	64.99	68.48
phrA	32.35	44.63	37.63	34.52	13.45	17.16	15.84	22.07
uvrC	5.07	6.08	5.76	7.68	6.43	8.87	6.58	8
uvrA	4.65	4.52	6.03	5.31	14.69	19.37	11.94	15.84
mutY2	4.04	3.5	4.44	4.46	5.13	6.58	6.28	11.23
mutY1	8.64	21.35	31.68	18.51	7.58	16.25	9.09	11.44
udg	17.15	16.82	24.53	18.13	31.84	50.3	37.13	38.7
alkA	0.54	0.97	1.61	1.98	2.58	3.89	1.67	3.33
xthA	5.31	6.43	4.7	3.77	7.01	7.47	5.81	7.26
mug	4.39	6.72	3.8	6.65	2.43	3.28	2.4	4.46
ruvC	2.65	2.99	3.31	3.8	4.96	4.16	2.94	3.64
ruvB	10.59	9.19	8.82	10.39	37.14	59.59	39.65	39.57
ruvA	1.33	1.02	0.6	1.16	5.56	4.96	2.12	4.61
ssb	44.19	54.35	52.2	51.15	70.93	59.19	59.36	82.04
xerD3	0.88	0.72	0.52	1.3	0.92	1.63	1.02	1.41
xerD2	0.53	0.08	0	0.71	0.25	0.59	0.36	1.26
xerD1	2.1	3.96	2.59	3.95	4.47	5.51	4.52	5.6
xerC	5.35	5.75	7.62	6.69	8.44	10.98	8.6	11.11
recR	171.23	194.98	205.3	174.14	150.54	328.35	259.77	246.07
recO	69.77	75.92	74.22	72.08	90.02	99.71	55.64	70.1
recN	7.6	8.35	10.41	9	20.73	16.12	15.33	16.49
recJ	3.06	3.11	5.03	3.99	10.49	10.6	7.37	8.92
recJ	6.7	6.36	7.46	7.72	16.55	11.99	11.66	12.08
recF	5.1	6.08	5.64	5.17	16.21	12.63	9.68	12.95
recA	264.09	299.88	269.16	275.11	572.22	595.86	487.01	617.22
clpB	23.35	25.42	28.54	28.93	47.52	40.23	29.37	37.87
clpP1	273.19	245.09	347.15	296.41	692.69	905.19	627.64	672.85
clpX	110.03	112.51	131.84	121.46	322.59	450.39	308.76	322.02
nusG	44.51	46.81	55.58	52.53	149.76	161.42	110.13	151.09

crhR	45.31	54.23	91.54	61.81	89.51	110.55	54.48	97.43
rpoA	75.02	76.13	85.11	85.63	193.99	263.86	117.25	195.65
smpB	9.95	10.08	13.85	14.03	16.21	18.27	13.05	16.32
rimO	189.99	214.86	316.25	273.63	146.22	196.1	119.05	179.16
fus	135.19	169.83	241.85	174.75	377.84	550.03	276.37	387.6
pfbB	65.5	61.55	69.48	68.78	155.69	223.86	150.07	167.26
tig	15.93	16.59	20.9	17.24	60.38	77.52	56.63	63.47
rplA	41.34	37.32	50.97	46.62	163.9	181.44	119.96	159.18
rplC	143.27	128.7	160.47	168.88	192.25	184.81	110.16	176.04
rplD	51.77	48.89	58.86	60.97	97.5	126.79	62.72	101.04
rplK	24.24	18.47	29.56	26.83	82.06	93.77	48.51	75.62
rplU	124.49	113.9	149.07	142.58	147.68	154.56	102.21	142.94
rplW	18.72	16.44	23.23	28.34	69.36	91.45	39.43	62.87
rpsL	2376.79	2550.22	2595.67	2560.35	870.37	1852.55	1059.69	1328.98
rpsU	175.18	168.43	192.27	207.63	206.52	193.47	140.87	211.12
ldh	0.18	0.22	0.16	0.7	0.29	0.76	0.3	0.67
ilvH	47.14	62.39	58.9	60.58	42.54	27.53	32.9	30.48
aroB	1.64	1.83	1.72	2.87	3.82	4.31	4.15	4.68
aroB	0.56	0.82	0.57	1.06	1.63	2.65	1.9	2.01
aroF1	9.54	6.59	10.53	10.57	28.62	33.35	23.62	28.06
aroF3	0.32	0.56	0.49	0.53	1.61	1.98	1.81	1.93
aroF4	13.75	4.77	6.81	13.8	21.63	15.79	18.32	20.18
trpA1	0.63	0.74	0.54	0.65	0.94	1.29	0.86	1.17
trpA2	3.54	4.28	3.36	4.94	7.43	11.75	6.97	9.17
trpC1	0.35	0.33	0.41	0.43	0.58	0.76	0.64	0.41
trpC2	8.81	7.3	9.29	8.68	40.98	34.36	21.85	30.74
trpD1	2.85	2.18	2.04	2.97	7.35	8.02	7.15	8.14
trpD2	4.65	4.72	4.08	5.53	16.46	15.25	9.19	11.9
trpE	0.62	0.76	0.7	0.73	0.82	0.88	0.81	1.12
tyrA2	1.26	1.63	1.52	1.63	2.02	3.23	2.05	2.32
cruA	2.38	2.77	2.61	3.42	7.75	8.47	6.02	7.23
cruG	8.05	8.92	8.11	9.28	10.92	8.72	6.69	8.54
cruG	0.53	0.37	0.32	0.86	1.35	1.67	0.73	1.47
cruH	1.29	1.36	1.47	1.78	2.22	1.77	1.71	2.53
cruP	2.26	2.88	2.66	3	3.59	3.52	2.62	3.77
crtB	6.62	6.56	5.34	6.78	8.31	8.06	5.59	7.77
crtE	5.46	4.81	5.64	5.57	27.25	23.87	17.38	22.14
crtH	2.79	2.3	3.38	2.85	8.12	9.25	6.19	9.52
crtP	12.3	13.48	14.86	13.55	26.9	23.08	22.44	24.39
crtQ	5.27	5.95	8.16	8.56	10.02	12.9	8.91	10.9
crtR	1.08	0.61	0.55	0.58	6.84	4.75	3.27	3.82
crtW	55.9	19.78	83.8	63.59	38.99	14.85	15.78	20.04
sodC	2.55	2.83	2.86	2.43	19.39	14.7	9.47	17.56

sodA2	1178.21	987.8	899.13	1103.99	899.62	652.82	594.82	655.98
katA	65.5	53.1	56.52	59.19	58.85	58.55	60.6	57.98
katG	130.38	187.83	78.72	94.6	338.26	246.55	223.95	317.92
PGdx	4.01	4.74	5.23	3.86	9.03	6.41	6.44	10.14
exoA	4.46	5.62	4.86	5.92	9.69	9.63	8.76	9.85
exoB	0.33	0.45	0.3	0.83	1.65	1.55	1.22	1.15
exoK	20.78	16.33	11.8	23.36	11.4	14.93	16.77	13.66
exoN	5.9	5.54	5.54	6.11	5.93	9.06	6.72	7.35
exoP	0.36	0.38	0.47	0.78	3.93	3.75	3.3	3.27
exoQ	11.71	10.18	10.48	13.31	21.08	19.08	13.99	17.21
exoT	43.56	32.83	45.25	43.91	171.11	167.3	128.91	158.3
exoV	1.48	1.84	1.51	2.55	4.93	4.03	2.75	4.42
wcaA	2.68	1.81	2.57	2.98	9.52	13.69	8.08	12.52
wcaD	11.71	10.18	10.48	13.31	21.08	19.08	13.99	17.21
wcaG	4.96	3	2.93	6.7	7.79	3.34	4.27	8.27
wcaJ	0.27	0.35	0.59	0.88	1.04	1.21	0.94	1.14
wcaL	0.17	0.28	0.05	0.44	0.97	1.3	0.75	1.3
wcaL	0.33	0.24	0.2	0.49	1.42	2.07	0.99	1.81
wcaL	0.18	0.57	0.17	1.24	5.79	3.18	2.82	4.6
wza	43.56	32.83	45.25	43.91	171.11	167.3	128.91	158.3
wzc	0.36	0.38	0.47	0.78	3.93	3.75	3.3	3.27
wzx	2.8	2.57	2.88	3.68	4.93	3.84	3.12	4.08

Table S1 Key gene expression data of flight and ground group samples after exposure to near space and rehydration. (The expression levels of genes were calculated using TPM value)
